# Epigenetic regulation in the inner ear and its potential roles in development, protection, and regeneration

**DOI:** 10.3389/fncel.2014.00446

**Published:** 2015-01-07

**Authors:** Wanda S. Layman, Jian Zuo

**Affiliations:** Department of Developmental Neurobiology, St. Jude Children’s Research HospitalMemphis, TN, USA

**Keywords:** hair cells, auditory, histone acetylation, histone methylation, DNA methylation, ototoxicity, histone deacetylase inhibitors, cellular reprogramming

## Abstract

The burgeoning field of epigenetics is beginning to make a significant impact on our understanding of tissue development, maintenance, and function. Epigenetic mechanisms regulate the structure and activity of the genome in response to intracellular and environmental cues that direct cell-type specific gene networks. The inner ear is comprised of highly specialized cell types with identical genomes that originate from a single totipotent zygote. During inner ear development specific combinations of transcription factors and epigenetic modifiers must function in a coordinated manner to establish and maintain cellular identity. These epigenetic regulatory mechanisms contribute to the maintenance of distinct chromatin states and cell-type specific gene expression patterns. In this review, we highlight emerging paradigms for epigenetic modifications related to inner ear development, and how epigenetics may have a significant role in hearing loss, protection, and regeneration.

## Introduction

In 1942, C. H. Waddington coined the term epigenetics by combining the words epigenesis and genetics (Waddington, 2012). Epigenesis refers to the sequence of events that occur during differentiation of cells from their initial totipotent state to a fully developed multicellular organism. The physical nature of genes and their role in heredity was unknown at the time Waddington coined this term. However, he used the term epigenetics to refer to the increasing irreversibility of cellular differentiation as a cell becomes more differentiated during development. The field of developmental biology continues striving to better understand how a single totipotent cell containing one genome has the ability to generate millions of highly specialized fully differentiated cells with very different gene regulatory networks.

The definition of epigenetics has evolved over the years and continues to be debated by scientists. Epigenetics was classically defined as heritable changes in gene function that cannot be explained by changes in DNA sequence and typically referred to DNA methylation related to parental genomic imprinting. However, this definition has changed drastically in recent years to encompass non-heritable changes that alter gene function including dynamic chromatin states regulated by various histone modifications and chromatin remodeling proteins. The development of the “omics era” has greatly impacted the way scientists now view epigenetics since many human developmental disorders and cancer have been correlated with the misregulation of specific epigenetic events and has driven the development of epigenetic therapeutics.

Although a vast amount of data about epigenetics exists for other tissues, there is a noticeable lack of information about epigenetic modifications in the inner ear. Proper epigenetic modifications are required for normal developmental processes. Gene expression must be coordinated in a temporal and cell-type specific manner and requires multiple levels of gene regulation. Although transcription factors are a primary source of gene regulation, epigenetic modifications regulate transcription factor access to target genes. This concept is apparent from studies looking at direct cellular reprogramming through ectopic expression of defined transcription factors, which show that direct reprogramming is a slow and inefficient process with most cells failing to reprogram (Huangfu et al., 2008; Mikkelsen et al., 2008). In the auditory field, ectopic expression of transcription factors such as *Atoh1* has been used to convert mammalian non-sensory epithelial cells into cells that express many endogenous hair cell markers (Zheng and Gao, 2000; Izumikawa et al., 2005; Gubbels et al., 2008). However, the reprogramming process of transforming supporting cells into hair cells may not be solely about genetic transformation, but also epigenetic transformation. Studies using induced pluripotent stem cells (iPSCs) have shown that they retain the epigenetic memory of their somatic cell of origin (Kim et al., 2010; Lister et al., 2011). The epigenetic memory retained by iPSCs can interfere with their potential for differentiation into other cell types (Li et al., 2009; Kim et al., 2010; Lister et al., 2011). Additionally, iPSCs derived from aged mice have a decreased potential for reprogramming compared to iPSCs derived from juvenile mice (Li et al., 2009; Lister et al., 2011). Although ectopic expression of transcription factors (*Atoh1*) can convert neonatal non-sensory epithelial cells into hair cell-like cells, loss of cellular plasticity at later postnatal ages could largely impact clinical application of this method (Kelly et al., 2012; Liu et al., 2012b).

In this review, we discuss the different types of epigenetic modifications and regulatory mechanisms in regards to development, disease, protection, and cellular reprogramming.

## Histone modifications

**Figure 1:**
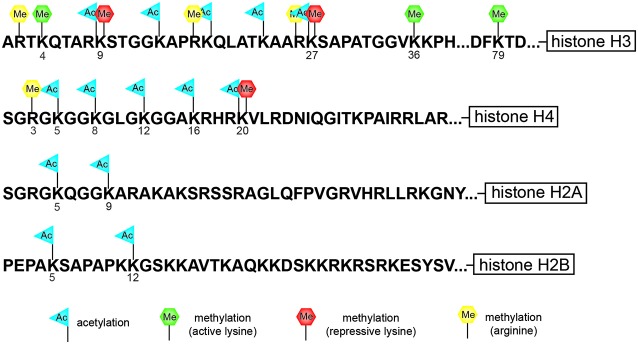
**Cartoon diagram indicates potential sites of modification at specific residues along the histones tail**. The tails of histone H3 and H4 have the largest number of potential modification sites including lysine (K)-specific methylation and acetylation sites and arginine (R)-specific methylation sites. The tail of histone H3 is subject to both repressive lysine (K)-specific methylation marks (K9 and K27) as well as activating lysine (K)-specific methylation marks (K4, K36, and K79).

Nucleosomes form the fundamental repeating units of eukaryotic chromatin, which is used to package large eukaryotic genomes into the nucleus while still ensuring appropriate access to the chromatin (Kornberg, 1974; Kornberg and Thomas, 1974). Nucleosomes are folded through a series of successively higher order structures to both compact DNA and create an added layer of regulatory control ensuring correct gene expression. The nucleosome is comprised of approximately 147 base pairs of DNA wrapped around eight histone core subunits consisting of two copies each of the core histones H2A, H2B, H3, and H4 (Luger et al., 1997). Histone H3 and H4 have long tails that protrude from the nucleosome and can be covalently modified at several sites (Figure 1; Vaquero et al., 2003; Campos and Reinberg, 2009; Bannister and Kouzarides, 2011). These histone tails are subjected to post-translational modification including acetylation, methylation, phosphorylation, ubiquitination, sumoylation, or ADP-ribosylation which is mediated by the counteracting activities of enzymes that add or remove such modifications (Vaquero et al., 2003; Campos and Reinberg, 2009; Bannister and Kouzarides, 2011).

### Histone acetylation

**Figure 2:**
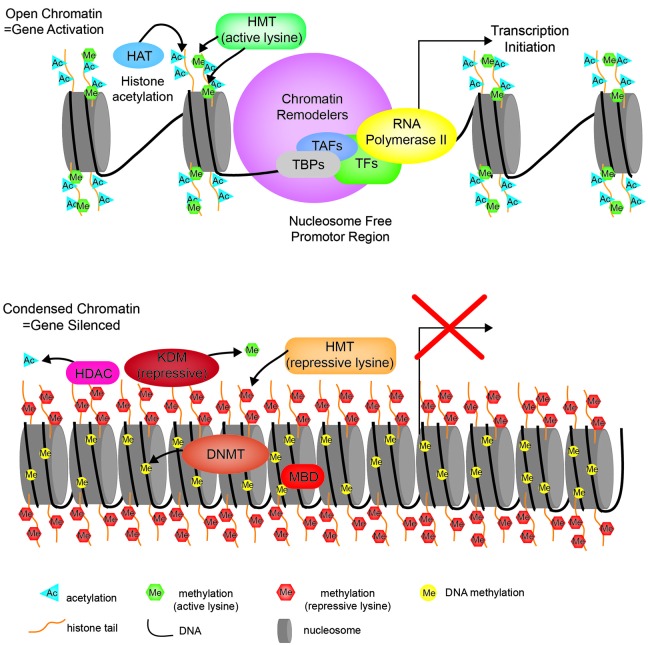
**Chromatin state.** Cartoon diagram depicting the open relaxed chromatin of an actively transcribed gene (upper portion), compared to the nucleosome dense compacted chromatin associated with a silenced gene (bottom portion). HMT—histone methyltransferase, HAT—histone acetyltransferase, TBP—TATA-binding protein, TAF—TBP-associated factors, TF—transcription factor, HDAC—histone deacetylase, KDM—lysine (K)-specific demethylase, DNMT—DNA methyltransferase, MBD—methyl-CpG-binding domain.

Posttranslational histone modifications alter histones interaction with DNA and nuclear proteins. Histone acetylation of specific lysine residues plays a fundamental role in transcriptional regulation. The enzymes responsible for maintaining proper histone acetylation states include histone acetyltransferases (HATs) and histone deacetylases (HDACs). Histones undergo acetylation to yield a more relaxed chromatin conformation resulting from a net change in the overall charge and reduced electrostatic interactions. HATs transfer the acetyl moiety from acetyl coenzyme A to specific lysine residues on the histone tail. Acetylated histones also act as a signal that recruits bromodomain-containing proteins, which are primarily transcription factors and cofactors to target genes activating their transcription (Zeng and Zhou, 2002). The HDACs act in opposition to the HATs by removing the acetyl groups from histone tails allowing histones to interact with DNA more tightly to form a compacted nucleosome structure. This increased rigidity of the chromatin prevents the incorporation of transcriptional machinery, effectively silencing gene transcription (Figure 2).

### Histone methylation and demethylation

**Figure 3:**
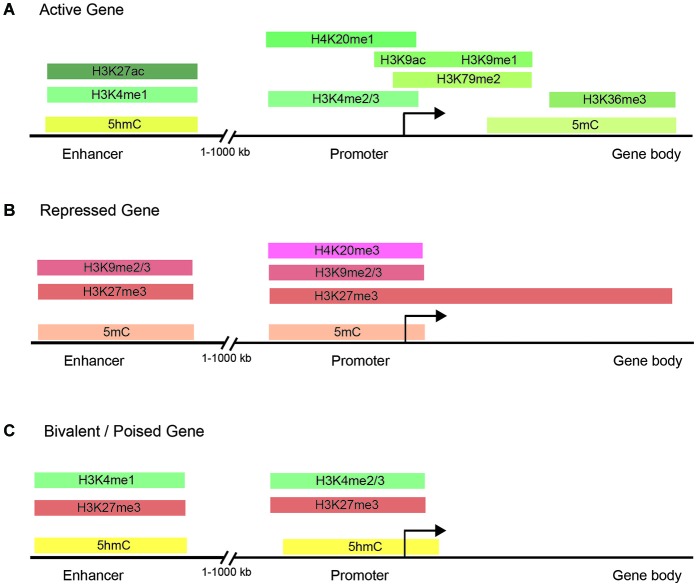
**Chromatin modifications are distributed in specific gene regulatory regions. (A)** The normal distribution of DNA methylation, DNA hydroxymethylation, and histone marks in the enhancer, promoter, and gene body of actively transcribed genes. Actively transcribed genes carry typically have chromatin modifications within the gene body to facilitate transcription initiation and elongation. **(B)** Common chromatin modifications found in the enhancer, promoter, and gene body of silenced genes. **(C)** Bivalent/poised genes have both activating and silencing chromatin modifications to facilitate rapid changes in gene expression during development.

Histone methylation, depending on both the histone and residue modified, contributes to either active or repressive chromatin configurations (Figure 2). Although histone methylation is not as well understood as acetylation, histones H3 and H4 are common methylation targets that can be methylated on arginine and lysine residues (Figure 1). While lysine can receive only one acetyl group, it can receive up to three methyl groups, and does not affect the overall charge of the residue. Specific histone methylation states serve as binding sites for the recruitment of additional regulatory proteins such as chromatin remodelers (Zhang and Dent, 2005; Wu and Zhang, 2009; Helin and Dhanak, 2013). As a general rule of thumb, sites of methylation that are typically associated with active gene transcription are H3K4me2/3 (promoter), H3K36me3 (3′ end gene body), and H3K79me2 (5′ end of gene body) (Azuara et al., 2006; Kolasinska-Zwierz et al., 2009; Onder et al., 2012; Fuchs et al., 2014). Whereas methylation marks associated typically with silenced genes are H3K9me2/3 (promoter and enhancer), H3K27me3 (promoter and enhancer), and H4K20me3 (promoter) (Azuara et al., 2006; Kolasinska-Zwierz et al., 2009). These common active and repressive histone marks are further illustrated in relation to the gene region in which they are detected in Figure 3.

Studies of histone methylation marks in mouse pluripotent embryonic stem cells (ESCs) have defined a class of developmentally regulated genes as “bivalent” since these genes are marked with both active (H3K4me3) and repressive (H3K27me3) histone modifications (Figure 3; Azuara et al., 2006; Bernstein et al., 2006; Mikkelsen et al., 2007; Voigt et al., 2013). By exhibiting both active and repressive features, bivalent genes are posited as being in a poised state, enabling them to be rapidly activated upon suitable developmental cues and/or environmental stimuli. Although bivalent genes were first described for ESCs, where the marks are most prevalent, later observations also detected bivalent domains in cell types of restricted potency such as neural progenitor cells (Mikkelsen et al., 2007; Rugg-Gunn et al., 2010).

The enzymes responsible for maintaining proper histone methylation status are histone lysine/arginine methyltransferases and histone lysine/arginine demethylases. Lysine-specific histone methyltransferases (HMTs) are subdivided into SET (**S**u(var)3–9, **E**nhancer of Zeste, **T**rithorax) domain-containing and non-SET domain-containing proteins. The argine-specific protein arginine methyltransferases (PRMTs) are responsible for methylating arginine residues on the histones. HMTs and PRMTs together have over 60 different family members all of which use S-Adenosyl methionine (SAM) as a cofactor and methyl donor group (Helin and Dhanak, 2013).

Histone methylation for many years was considered to be a permanent and irreversible histone modification due to the low turnover rate of methylated histones (Byvoet et al., 1972). However, the discovery of lysine specific demethylase 1 (LSD1, officially known as KDM1A) and later the JmjC-domain-containing lysine demethylase family has completely changed this view (Kooistra and Helin, 2012). Interestingly, LSD1 can catalyze the demethylation of H3K4me1/2 and H3K9me1/2, which means that LSD1 has the ability to both silence and activate gene transcription (Zhang and Dent, 2005; Wu and Zhang, 2009; Helin and Dhanak, 2013). LSD1 is also reported to demethylate non-histone target proteins such as p53, DNMT1, and E2F1 (Huang et al., 2007; Wang et al., 2009; Kontaki and Talianidis, 2010; Helin and Dhanak, 2013; Mosammaparast et al., 2013). LSD1 and its close relative LSD2 belong to the superfamily of flavin adenine dinucleotide (FAD)-dependent monooxidases. Unlike the LSD demethylases, the JmjC-domain-containing demethylases can also demethylate trimethylated lysines. This catalysis involves an oxidative mechanism requiring iron and 2-oxoglutarate as co-factors and is posited to occur through direct hydroxylation of the affected methyl group.

### Histone variants

Although histones have a conserved role as general DNA packaging agents, it has become clear that another key function of these proteins is to confer variation in chromatin structure to ensure dynamic patterns of transcriptional regulation. Some histone variants have distinct biophysical characteristics that are thought to alter the properties of nucleosomes, while other variants are targeted to specific regions of the genome. Specific histone variants are exchanged with the pre-existing histones during critical periods of development and differentiation (Brandt et al., 1979; Grove and Zweidler, 1984; Wunsch et al., 1991; Bosch and Suau, 1995). This replacement can often result in the variants becoming the predominant species in the differentiated cell (Pina and Suau, 1987; Wunsch et al., 1991). Mutations in specific histone variants and their associated chaperone machinery contribute to human disease such as cancer (Maze et al., 2014), suggesting an essential function for regulation of histone variants during specific aspects of cellular differentiation.

## DNA methylation

Methylation at the 5-positon of cytosine (5-mC) is one of the best studied and most mechanistically understood epigenetic modifications that is well conserved among most plant, animal and fungal models (Feng et al., 2010). Three conserved DNA methyltransferase enzymes, DNA methyltransferase 1 (DNMT1), DNMT3A and DNMT3B, are responsible for the deposition and maintenance of DNA methylation and are essential for normal development (Li et al., 1992; Okano et al., 1999). Mammalian genomes are globally CpG-depleted and roughly 60–80% of the 28 million CpGs in the human genome are generally methylated (Smith and Meissner, 2013). Less than 10% of CpGs occur in the CG-dense regions called CpG islands (Deaton and Bird, 2011). CpG islands are prevalent at transcription start sites (TSSs) of housekeeping genes and genes involved in developmental processes (Deaton and Bird, 2011). Most genomic DNA methylation patterns are static across tissues and throughout life and only change in localized contexts as specific cellular processes are activated or repressed (Figure 3). However, the exception to this is in the germ line and during pre-implantation embryonic development, when DNA methylation levels are globally reset (Smith and Meissner, 2013).

Numerous assays have been developed since DNA methylation was originally postulated as an epigenetic regulator to study cytosine methylation (Holliday and Pugh, 1975; Riggs, 1975). This includes assays such as methylation-sensitive restriction enzyme mapping, deamination of unmethylated cytosines with sodium bisulphite, and targeting methylated DNA directly using antibodies for enrichment. High-throughput sequencing has enabled complete methylomes to be elucidated, such that methylation sites are now mapped at base-pair resolution across development from zygote to terminally differentiated adult cells. However, global DNA methylation patterns during development and aging are tissue and cell type-specific (Calvanese et al., 2012).

DNMT1 is critical for maintaining DNA methylation during mitosis. During DNA replication, DNMT1 is positioned at the replication fork and transfers the methylation marks to the newly synthesized daughter strand and is essential for stable repression of genes after cell division. Although DNA methylation maintenance ensures epigenetic inheritance at established positions, there are many instances in which methylation must be specifically targeted and others in which methylation must be inhibited or removed. DNMT3A and DNMT3B are responsible for establishing *de novo* DNA methylation, primarily at CpG dinucleotides (Jurkowska et al., 2011). DNMT3A and DNMT3B target promoters in complex with other epigenetic repressors, including HDACs and repressive HMTs such as EZH2 and G9a. Additionally, crosstalk exists between some site-specific transcription factors and DNMTs for example DNMT1, DNMT3A, and DNMT3B have been shown to interact with transcription factors such as E2F1, E2F6, and Atoh1 to facilitate or prevent DNA methylation at specific target genes (Robertson et al., 2000; Bossuyt et al., 2009; Velasco et al., 2010). Loss of all three DNMTs in ESCs does not affect their survival or stem cell molecular identity, but the ability to differentiate is completely inhibited (Jackson et al., 2004; Tsumura et al., 2006). ESCs lacking DNA methylation fail to up-regulate germ layer associated markers and are unable to efficiently silence pluripotency genes (Jackson et al., 2004). Typically the transcriptional network associated with pluripotency is rapidly silenced upon differentiation through both maintenance and *de novo* methylation, since embryonic programs must be resolved towards specific cell lineages.

5-mC was initially the only known DNA-specific epigenetic mark, then in 2009, 5-hydroxymethylcytosine (5-hmC) was discovered as another relatively abundant cytosine modification in mouse Purkinje neurons and ESCs (Kriaucionis and Heintz, 2009; Tahiliani et al., 2009). The ten-eleven translocation (TET) proteins mediate the oxidation of 5-mC to 5-hmC (Tahiliani et al., 2009; Wang et al., 2012), which is then further oxidized in a stepwise manner to 5-formylcytosine (5-fC) and 5-carboxylcytosine (5-caC; He et al., 2011; Ito et al., 2011). An emerging finding is that 5-mC and 5-hmC are dynamically regulated both within and across cell types (Kriaucionis and Heintz, 2009; Tahiliani et al., 2009; Szulwach et al., 2011; Shen and Zhang, 2013). Although 5-hmC may simply act as a DNA demethylation intermediate, studies have shown that 5-hmC not only impairs the binding of 5-mC binding proteins (Valinluck et al., 2004), but also has its own unique binding protein, MBD3, (Yildirim et al., 2011) and shows unique distribution patterns in the genome (Stroud et al., 2011; Szulwach et al., 2011). 5-hmC is enriched in gene dense euchromatic regions, and particularly at TSSs, promoters, and enhancers (Shen and Zhang, 2013). Additionally, 5-hmC is specifically enriched at gene promoters associated with bivalent domains marked with both the permissive mark H3K4me2/3 and the repressive mark H3K27me3, but is absent from heterochromatin marked by H3K9me3 (Shen and Zhang, 2013).

Recent genome-wide analysis of DNA methylation in human cells has identified a widespread distribution of 5-mC and, paradoxically, has shown hypermethylation in the gene bodies of actively transcribed genes (Figure 3; Lister et al., 2009; Stadler et al., 2011; Hon et al., 2013; Ziller et al., 2013) and hypomethylation was found at active enhancers (Lister et al., 2009; Stadler et al., 2011; Hon et al., 2013; Ziller et al., 2013). 5-hmC is also significantly enriched at distal cis-regulatory sequences, suggesting that dynamic DNA methylation at these regions is likely mediated by interplays between DNMT mediated methylation and TET mediated demethylation processes (Stroud et al., 2011; Szulwach et al., 2011; Yu et al., 2012). Together, these studies have underscored the diverse roles that DNA methylation has in gene regulation and the need for systematic mapping and characterization of DNA methylomes in different tissues and cell types during development and aging. Since proper maintenance of 5-mC and 5-hmC by DNMT and TET proteins has been shown to be critical for proper neurodevelopment and memory (Wang et al., 2012), aberrant alterations in DNA methylation are also correlated with diseases such as diabetes, schizophrenia, multiple sclerosis, cancer, and cellular senescence (Jurkowska et al., 2011).

## Chromatin remodelers

At least three processes control the assembly and regulation of chromatin: histone modifications, DNA methylation, and ATP-dependent chromatin remodeling. ATP-dependent chromatin remodelers alter the physical state of chromatin by either sliding nucleosomes in relation to the DNA or exchanging nucleosomes into and out of DNA. Chromatin remodelers act as “readers” of the histone modifications to regulate chromatin structure and gene expression. Approximately 30 genes encode the ATP-dependent chromatin remodeling subunits in mammals. With few exceptions, the ATP-dependent chromatin remodeling proteins appear to be genetically non-redundant.

Mutations in ATP-dependent chromatin remodeling genes often have severe effects on the early embryo or give rise to maternal-effect phenotypes in which the phenotype of the embryo reflects the genotype of the mother. In many cases, the genes encoding the ATP-dependent chromatin remodeling proteins or their subunits are haploinsufficient, which indicates that their role in specific developmental processes is likely rate limiting. For instance, heterozygous mutation in the chromodomain helicase DNA binding protein 7 (CHD7) causes CHARGE syndrome, a multiple anomaly disorder that is a common cause of deaf-blindness in humans (Zentner et al., 2010), whereas duplication or overexpression of *CHD7* is associated with multiple forms of cancer including colorectal cancer, pancreatic cancer, small-cell lung cancer, and gastric cancer (Pleasance et al., 2010; Kim et al., 2011; Colbert et al., 2014; Tahara et al., 2014). These data together suggest that many cell types may be highly sensitive to chromatin remodeler dosage during development and aging.

## Epigenetics and hearing loss

Hereditary hearing loss or deafness has been associated with mutations in genes whose proteins regulate the chromatin state and include genes involved in DNA methylation, histone modification and chromatin remodeling (Table 1). Autosomal dominant cerebellar ataxia, deafness, and narcolepsy (ADCADN) and hereditary sensory neuropathy type IE (HSN1E) are both caused by heterozygous mutation in the DNMT1 gene (Sun et al., 2014). ADCADN is characterized by late onset (age 30–40 years) narcolepsy–cataplexy, sensorineural deafness, cerebellar ataxia, dementia, psychosis, optic atrophy, and other symptoms (Winkelmann et al., 2012). Narcolepsy and deafness are typically the first symptoms to appear followed by ataxia (Winkelmann et al., 2012). People with HSN1E develop hearing loss that is caused by abnormalities in the inner ear leading to sensorineural hearing loss (Wright and Dyck, 1995; Hojo et al., 1999; Klein et al., 2011). Hearing loss worsens over time and usually progresses to moderate or severe deafness between the ages of 20 and 35 (Wright and Dyck, 1995; Hojo et al., 1999; Klein et al., 2011). Mutations in DNMT1 typically cause bilateral hearing loss but unilateral hearing loss has also been reported (Melberg et al., 1995).

**Table 1:** **Epigenetic factors associated with hearing loss in humans**.

Human disease	Gene	Type of hearing loss
	*DNA methyltransferase*			
Autosomal dominant cerebellar ataxia, deafness, and narcolepsy (ADCADN)	DNMT1	Sensorineural
Hereditary sensory neuropathy type IE (HSN1E)	DNMT1	Sensorineural
	*Histone methyltransferase*		
Sotos syndrome	NSD1	Conductive
Weaver syndrome	EZH2	Conductive
Kleefstra syndrome	EHMT1	Sensorineural,
Kabuki syndrome	KMT2D	Sensorineural Conductive, or Mixed
	*Histone acetyltransferase*				
Say-Barber-Biesecker variant of Ohdo syndrome	KAT6B	Sensorineural
Genitopatellar syndrome (GPS)	KAT6B	Sensorineural
	Chromatin remodeler		
CHARGE syndrome	CHD7	Sensorineural

*Human diseases associated with hereditary hearing loss or deafness caused by heterozygous mutation in genes whose protein product is involved in DNA methylation, histone modification, or chromatin remodeling*.

Mutations in HMT have also been associated with hearing loss. Overgrowth disorders, Sotos syndrome and Weaver syndrome, are caused by heterozygous mutations in the HMT NSD1 (Sotos and Weaver syndromes) and EZH2 (Weaver syndrome) (Tatton-Brown and Rahman, 2013; Tatton-Brown et al., 2013). Sotos syndrome is frequently associated with conductive hearing loss while only a few cases of hearing loss have been reported for Weaver syndrome (Tatton-Brown et al., 2013). Although a large degree of phenotypic overlap exists between Sotos syndrome and Weaver syndrome, NSD1 and EZH2 regulate different methylation sites. NSD1 has been shown to preferentially dimethylate H3K36 and H4K20, the impact of dimethylation at either of these two lysine residues is not entirely clear at this time (Qiao et al., 2011). However, EZH2 has been well characterized as it functions as the catalytic subunit for the polycomb repressive complex 2 (PRC2) and is a critical regulator of H3K27me3.

Kleefstra syndrome is a developmental disorder characterized by intellectual disability, childhood hypotonia, distinctive facial features and sensorineural hearing loss (Kleefstra et al., 2005). Heterozygous mutation in euchromatic histone-lysine N-methyltransferase 1 (EHMT1) is causative for Kleefstra syndrome (Kleefstra et al., 2005). EHMT1 is part of the E2F6 complex which represses transcription via methylation of histone H3K9 (Tachibana et al., 2005).

Heterozygous mutation in lysine (K)-specific methyltransferase 2D (KMT2D, also known as MLL2/MLL4) is associated with Kabuki syndrome (Ng et al., 2010). Kabuki syndrome is characterized by distinctive facial features, mild to moderate mental impairment, microcephaly, hypertonia, skeletal abnormalities, heart abnormalities, and hearing loss (Ng et al., 2010). Hearing loss is a common finding in Kabuki syndrome and can be conductive, sensorineural or mixed (Barozzi et al., 2009). KMT2D is a histone methyltransferase that has been shown to mono- and dimethylate H3K4 (Lee et al., 2013). Approximately 6 percent of Kabuki syndrome cases are caused by heterozygous mutation in lysine (K)-specific demethylase 6A (KDM6A). KDM6A catalyzes the demethylation of H3K27me3. Although, KMT2D and KDM6A have different functions at different lysine residues, both ultimately act as transcriptional activators (Jiang et al., 2013; Lee et al., 2013).

Heterozygous mutations in lysine acetyltransferase 6B (KAT6B, also known as MYST4) are associated with both genitopatellar syndrome (GPS) and Say-Barber-Biesecker variant of Ohdo syndrome (Campeau et al., 2012a; Simpson et al., 2012). These KAT6B-related disorders have phenotypic overlap that includes significant global developmental delay/intellectual disability, hypotonia, cryptorchidism, patellar hypoplasia/agenesis, congenital heart defects, dental anomalies, hearing loss, and thyroid anomalies (Campeau et al., 2012a; Simpson et al., 2012). KAT6B is a HAT that also has transcriptional activation activity in the N-terminal end of the protein and transcriptional repression activity in the C-terminal end of the protein (Campeau et al., 2012b). Mutations leading to GPS occur in the proximal portion of the last exon and lead to the expression of a protein without a C-terminal domain, while mutations leading to Say-Barber-Biesecker variant of Ohdo syndrome occur either throughout the gene, leading to nonsense-mediated decay, or more distally in the last exon (Campeau et al., 2012b).

CHD7 has been more extensively characterized in regards to inner ear development than the epigenetic factors listed above. CHD7 haploinsufficiency causes CHARGE syndrome, the most consistent clinical feature associated with CHARGE is inner ear defects, including semicircular canal dysplasia that typically affects all three canals, and a Mondini form of cochlear hypoplasia (Layman et al., 2010; Zentner et al., 2010). Inner ear phenotypes observed in mouse models of CHARGE syndrome are similar to those reported in CHARGE patients and include semicircular canal defects, innervation defects, and vestibular dysfunction (Kiernan et al., 2002; Hawker et al., 2005; Adams et al., 2007; Hurd et al., 2007). Complete absence of CHD7 results in decreased expression of patterning and pro-neural genes including *Otx2, Fgf10, Ngn1, NeuroD*, *Islet1, Rarb*, and *Rxrg* genes leading to reduced proliferation of developing neuroblasts and inner ear malformations (Hurd et al., 2010; Micucci et al., 2014). Additionally, a recent report found that CHD7 deficiency triggers both p53 expression and activation (Van Nostrand et al., 2014). These data together provide a basis for understanding how CHD7 deficiency results in the profound yet variable phenotypes associated with CHARGE syndrome and potential targets for therapeutic development.

## Epigenetics: damage and protection

Auditory hair cells have repeatedly been shown to be susceptible to ototoxicity from a multitude of drugs including aminoglycoside antibiotics such as gentamicin, loop diuretics such as furosemide, platinum-based chemotherapy agents such as cisplatin, and a number of non-steroidal anti-inflammatory drugs (NSAIDS). Additionally, noise is one of the most common causes of hearing loss, and one of the most common occupational illnesses in the United States. The formation of reactive oxygen species (ROS) is the major cause that underlies the molecular pathology of hair cell death related to noise induced trauma as well as aminoglycoside antibiotic and cisplatin treatment (Cheng et al., 2005; Schacht et al., 2012). ROS production is associated with increased DNA damage and chromosomal degradation with alterations of both hypermethylation and hypomethylation of the DNA (Campos et al., 2007; Lim et al., 2008; Donkena et al., 2010; Ziech et al., 2010, 2011). Aminoglycoside antibiotics have also been shown to cause increased histone deacetylation in mammalian hair cells through recruitment of HDACs to the chromatin (Jiang et al., 2006; Chen et al., 2009).

HDAC inhibitors were originally used as anti-cancer agents and some are approved by the FDA for use in the treatment of specific types of cancer in humans. However, broad spectrum and HDAC-specific inhibitors are also known to have protective effects in a concentration dependent manner in inflammation, neurodegeneration, and oxidative stress models (Ryu et al., 2003; Liu et al., 2012a; Robert and Rassool, 2012). HDAC inhibitors are primarily thought to modulate chromatin condensation by regulating histone acetylation and thus affect gene expression. HDAC inhibitors have also been shown to affect the post-translational modification of some important intracellular non-histone proteins, such as heat shock protein 90 and Rel-A/p65 (Yu et al., 2002; Faraco et al., 2009). In the inner ear, HDAC inhibitors also have a protective effect on hair cells subjected to aminoglycosides *in vitro* (Chen et al., 2009) and cisplatin *in vivo* (Drottar et al., 2006). However, the precise mechanism underlying their protective effect in the inner ear remains unknown.

## Cellular reprogramming and hair cell regeneration

Reprogramming cell fate through transcription factor(s) over-expression is a general and powerful approach for regenerative medicine (Cohen and Melton, 2011). However, direct reprogramming through ectopic expression of defined transcription factors is a slow and inefficient process that requires weeks, with most cells failing to reprogram (Huangfu et al., 2008; Mikkelsen et al., 2008). Additionally, the efficiency and yield of cellular reprogramming rapidly declines with increasing age and differentiation status of the donor cell (Hanna et al., 2010; Kim et al., 2010; Lister et al., 2011). A large reconfiguration of the chromatin structure, from DNA methylation to histone modifications and nucleosome remodeling, occurs during somatic cell reprogramming to a pluripotent state. These layers of epigenetic regulation are often used as repressive mechanisms in somatic cells to prevent unwanted gene expression from other lineages. How these epigenetic barriers to reprogramming are overcome is a key question, since the epigenetic memory of the somatic cell largely impacts its capacity for cellular reprogramming. Several lines of evidence support the notion that the process of reprogramming involves rare stochastic epigenetic events. Studies have shown that inhibitors of epigenetic events such as DNA methylation, histone deacetylation, and histone methylation are able to improve reprogramming efficiency (Huangfu et al., 2008; Mikkelsen et al., 2008; Hanna et al., 2010; Kim et al., 2010; Lister et al., 2011).

The epigenetic modifications made during inner ear development remain mostly unknown at this time. However, cofactors of repressive complexes such as NuRD and PRC2 have been reported to be present in the neonatal mouse organ of Corti. The NuRD cofactors including LSD1 are present throughout most of the organ of Corti from E18.5 until P4, then completely absent by P7, and are detectable again from P8 through P21 (Layman et al., 2013). The PRC2 enzymatic subunit, EZH2 is also highly present from E18.5 to P0 in the mouse organ of Corti, absent between P2 and P4, and is evident again throughout the organ of Corti by P6 and persists through P21 (Layman et al., 2013). The presence of these repressive complexes also correlates with transcriptional silencing of known target genes of LSD1 and EZH2 including genes required for proliferation (mTert) and cell fate specification (Atoh1), which is consistent with reports related to organ of Corti quiescence and maturation during neonatal development. Additionally, DNMT3A and DNMT3B are also reported to have a dramatic increase in expression after the first postnatal week in the mouse organ of Corti (Mutai et al., 2009; Layman et al., 2013). DNA methylation is one of the most common and irreversible epigenetic modifications that control gene expression. DNA methylation may repress genes encoding drug metabolizing enzymes, drug transporters, or even drug target genes, which may alter the pharmacokinetics and pharmacodynamics of drugs that may be ototoxic or drugs designed to facilitate hearing regeneration. A better understanding of how the genes in the inner ear are regulated epigenetically will allow researchers the ability to design therapeutic agents that may bypass or alter the chromatin state making it more amenable to cellular reprogramming.

Unlike mammalian hair cells, hair cells in the avian basilar papilla and utricle are rapidly regenerated after ototoxic injury (Corwin and Oberholtzer, 1997). New avian hair cells are generated from the epithelial supporting cells through renewed supporting cell proliferation and by direct cellular conversion from a supporting cell to a hair cell (Raphael, 1992; Weisleder and Rubel, 1993; Roberson et al., 2004). However, pharmacological inhibition of HDACs results in decreased proliferation of avian vestibular supporting cells, both in dissociated culture and in intact utricles (Slattery et al., 2009). The reduction in supporting cell proliferation causes a reduction in the number of regenerated hair cells but does not directly affect hair cell differentiation (Slattery et al., 2009). These data indicate that HDACs have a critical function in regulating gene expression in non-sensory epithelial cells responding to ototoxic insult during normal regenerative processes. Further analysis is needed to determine which genes are being regulated by HDACs and whether each specific gene is critical for the proliferative response in supporting cells. Naturally regenerating systems such as the avian basilar papilla and utricle provide much needed information about the regulatory networks that are required for hair cell regeneration. Ideally, a comparison of the DNA methylome and histone modifications between the naturally regenerating system and the mammalian system following ototoxic insult would provide vital information about the types of epigenetic events that must occur to achieve complete mammalian hair cell regeneration.

The cell type specific distribution of histone modifications, DNA methylation, and chromatin remodeling events needs to be characterized during inner ear development from a multipotent progenitor cell to a terminally differentiated cell. The mammalian inner ear offers unique challenges for evaluating the epigenome given the limited number of cells across a diversity of cell types. Transcriptomic analysis of the mammalian inner ear during development by microarray or RNA-seq may provide a starting point for analyzing epigenetic modifications that may regulate the expression of specific target genes and microRNAs (Elkan-Miller et al., 2011; Smeti et al., 2012; Rudnicki et al., 2014). Emerging evidence has shown that more than one hundred microRNAs are regulated by epigenetic mechanisms, and about 50% of them are modulated by DNA methylation (Saito et al., 2006; Kunej et al., 2011; Suzuki et al., 2011). Additionally, a subgroup of microRNAs has been shown to directly target the enzymatic effectors of epigenetic modifications, which adds more complexity to the epigenetic regulatory network (Garzon et al., 2009; Iorio et al., 2010).

## Conclusions

Given that epigenetics is a cornerstone of development and cellular reprogramming, it seems likely that understanding and manipulating the epigenome holds enormous promise for preventing and treating hearing loss in humans. Epigenetics also offers an important window to understanding how ototoxic compounds and noise affect gene regulatory networks and how these epigenetic modifications may be manipulated and overcome utilizing epigenetic therapeutics. Understanding epigenetic mechanisms has become a major focus for research in most biological systems. The field of hearing research could greatly benefit from the vast amounts of information that can be garnered from epigenetic work in other biological systems to gain a better understanding of the complex gene regulatory networks being regulated in the inner ear.

## Conflict of interest statement

The authors declare that the research was conducted in the absence of any commercial or financial relationships that could be construed as a potential conflict of interest.
